# Genomic Instability of iPSCs: Challenges Towards Their Clinical Applications

**DOI:** 10.1007/s12015-016-9680-6

**Published:** 2016-09-05

**Authors:** Masahito Yoshihara, Yoshihide Hayashizaki, Yasuhiro Murakawa

**Affiliations:** 1 Division of Genomic Technologies, RIKEN Center for Life Science Technologies, Yokohama, Kanagawa Japan; 2 Department of Ophthalmology, Osaka University Graduate School of Medicine, Suita, Osaka Japan; 3 RIKEN Preventive Medicine and Diagnosis Innovation Program, Wako, Saitama Japan

**Keywords:** iPSCs, Genomic instability, Mutation, Regenerative medicine, Clinical application

## Abstract

Induced pluripotent stem cells (iPSCs) are a type of pluripotent stem cells generated directly from mature cells through the introduction of key transcription factors. iPSCs can be propagated and differentiated into many cell types in the human body, holding enormous potential in the field of regenerative medicine. However, genomic instability of iPSCs has been reported with the advent of high-throughput technologies such as next-generation sequencing. The presence of genetic variations in iPSCs has raised serious safety concerns, hampering the advancement of iPSC-based novel therapies. Here we summarize our current knowledge on genomic instability of iPSCs, with a particular focus on types of genetic variations and their origins. Importantly, it remains elusive whether genetic variations in iPSCs can be an actual risk factor for adverse effects including malignant outgrowth. Furthermore, we discuss novel approaches to generate iPSCs with fewer genetic variations. Lastly, we outline the safety issues and monitoring strategies of iPSCs in clinical settings.

## Introduction

Induced pluripotent stem cells (iPSCs) can be generated directly from patient-derived somatic cells by introducing defined sets of key transcription factors [1–3]. iPSCs can be potentially differentiated into many cell types in our body. Thus, iPSCs can be used as a powerful tool for disease modeling, pharmacological screening, and regenerative medicine for a wide range of diseases (see Avior et al. [4] and Robinton & Daley [5] for review). There are several advantages to iPSCs over pre-existing pluripotent stem cells. Importantly, iPSCs have solved the ethical issue of embryonic stem cells (ESCs) because iPSCs can be generated without destructing pre-implantation stage embryos. Furthermore, iPSC technologies have made it feasible to create patient-matched pluripotent stem cells. Differentiated cells derived from iPSCs are unlikely to cause immune rejection after transplantation [6, 7].

In 2014, the first-in-human clinical trial of iPSC-based cell therapy was conducted. A Japanese woman with exudative age-related macular degeneration (AMD) was implanted with a retinal pigment epithelial cell (RPE) sheet, which was differentiated from iPSCs generated from her own skin fibroblasts [8, 9]. Clinical application of iPSC-based novel therapies will give hope to patients suffering from intractable diseases.

However, recent reports on genomic instability of iPSCs have raised serious safety concerns with respect to tumorigenicity. In fact, genetic mutations were identified in the iPSCs which were supposed to be used in the second human clinical trial of iPSC-based therapy in 2015 [9, 10]. Even though there was no clear evidence that these mutations could directly lead to adverse effects, the planned transplantation surgery of iPSC-derived RPE sheet was cancelled [9, 10].

To facilitate the advancement of iPSC-based novel therapies, it is important to gain a deeper understanding of how and when these mutations occur. Furthermore, it is crucial to elucidate whether these mutations could actually confer harmful effects.

Here we summarize current understanding on the genomic instability of iPSCs. We discuss the characteristics of genetic variations in iPSCs, particularly focusing on their origins and their functional consequences. Finally, we outline the safety issues of iPSC-based cell therapies, and further discuss how to monitor and reduce genomic instability of iPSCs.

## Genomic Instability in iPSCs

In this section, we first introduce methods to detect genomic instability of iPSCs, and then describe each type of genetic variations identified in iPSCs using these methods.

### Methods for Detection of Genomic Instability

A number of technologies have been developed to detect genomic aberrations or mutations on a genome-wide scale. One of the most conventional methods is Giemsa (G)-banding, which can detect numerical (aneuploidy and polyploidy) or large structural chromosomal changes including translocations and inversions [11]. G-banding is readily applicable and is most widely used for genetic evaluation [12]. To achieve higher resolution, array-based technologies such as comparative genomic hybridization (CGH) [13] and single nucleotide polymorphism (SNP) arrays [14] have been adopted. These technologies allow us to investigate copy number variations (CNVs) (i.e., duplications and deletions) across the whole genome at kilobase resolution (for review see Le Scouarnec & Gribble [15]). However, these array-based methods cannot accurately detect balanced translocations and inversions [16]. Recently, the advent of next-generation sequencing (NGS) has enabled us to detect (i) genetic variations across the entire genome at single nucleotide resolution [17] and (ii) low frequency variations which could not be identified by conventional methods [18], revolutionizing the field of genomic research including genomic studies of iPSCs.

### Chromosomal Aberration

Chromosomal instability of human iPSCs was first reported in 2010 [19]. A large-scale study [20] as well as several individual studies [19, 21, 22] have investigated chromosomal aberrations in both human ESCs and human iPSCs, and reported that trisomy 12 is most recurrently observed in both cell types. Because chromosome 12 contains cell cycle-related genes and harbors pluripotency-associated gene *NANOG* [19], trisomy 12 might contribute to the selective advantage of proliferation and reprogramming in pluripotent stem cells. Mayshar et al. also reported that a gain of the 12p region was caused by prolonged culture [19]. Interestingly, the gain of 12p is a hallmark of testicular germ cell tumors [23, 24]. Other frequently recurrent aneuploidies in both cell types are amplifications of chromosome 8 and X [20]. In addition, frequencies of chromosomal aberrations were not remarkably different between human iPSCs and ESCs [20]. Although many common chromosomal aberrations are reported, different types of chromosomal aberrations are also identified [19, 20]. The reason for these differences remains to be elucidated (for review see Lund et al. [25]).

### Copy Number Variation

The first CNV analysis of human iPSCs was conducted by Chin et al. using array CGH [26]. Chin et al. found a few CNVs in each iPSC line, but none of the CNVs were shared between iPSC lines [26]. Several larger-scale studies later identified an amplification of 20q11.21 as the most recurrent CNV hotspot [21, 22, 27]. This CNV was also found in human ESCs [21, 22, 27]. Duplication of 20q11 is also frequently found in several cancer types [28, 29]. This region is enriched with genes associated with pluripotency and anti-apoptosis, such as DNA methyltransferase 3B (*DNMT3B*), inhibitor of DNA binding 1 (*ID1*), and BCL2-like1 (*BCL2L1*).

Furthermore, Laurent et al. [22] and Hussein et al. [30] analyzed the dynamic changes of CNVs during human iPSC passages using SNP array, and identified a large number of CNVs in early passage iPSCs [22, 30]. Interestingly, the number of CNVs decreased during cell passages [30]. These observations imply that CNVs are generated during reprogramming and that the mosaicism is gradually lost during cell passaging as a result of selective pressure. Most CNVs observed in early passage were deletions, which might be disadvantageous to cell growth or survival [30]. Laurent et al. reported that deletions of tumor-suppressor genes are frequently observed in early-passage human iPSCs but that duplications of oncogenic genes increase during cell passages [22].

A more recent whole genome sequencing (WGS) analysis reported that at least half of the CNVs observed in iPSCs are derived from low frequency somatic variants in the parental skin fibroblasts [31]. This discrepancy might be due to the limited dynamic range of array-based detection of low frequency CNVs in parental somatic cells (see review by Liang & Zhang [32]). NGS technologies have enabled us to detect such low frequency variations at single nucleotide resolution, providing deeper insights into the origin of genomic instability.

### Single Nucleotide Variant

Single nucleotide variants (SNVs) in iPSCs have been investigated by high-throughput NGS analysis such as WGS or whole exome sequencing (WES). These studies identified an average of ~10 protein-coding mutations per human iPSC line [33–37]. So far, recurrent SNVs have been rarely reported, but larger studies are still needed for comprehensive profiling of SNVs in iPSCs.

**Fig. 1:**
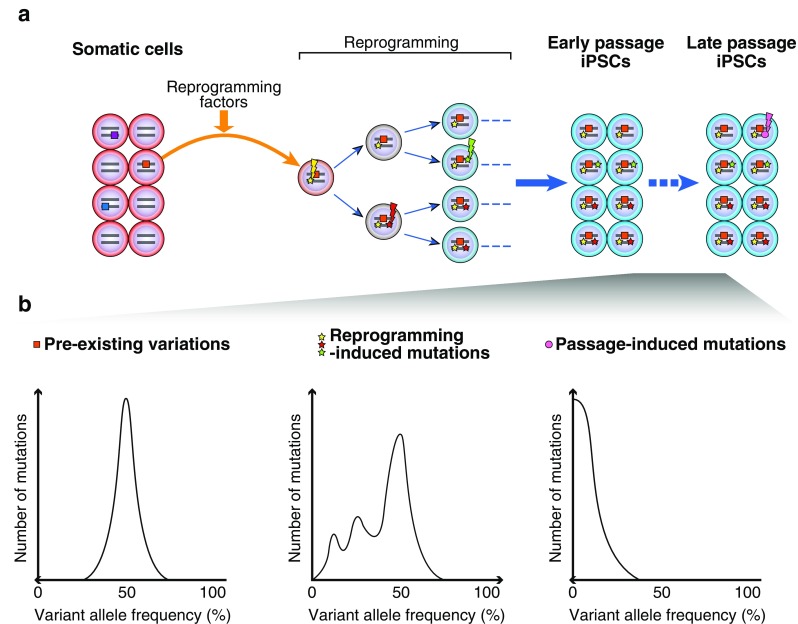
Origin of genetic variations in iPSCs. (**a**) Genetic variations of iPSCs have at least three origins: (i) pre-existingvariations in parental somatic cells, which can be manifested by a cloning procedure during iPSC generation, (ii) reprogramming-induced mutations which occur during the reprogramming process, and (iii) passage-induced mutations which arise during the prolonged culture. **(b)** (Left) Pre-existing variations (square) that exist in a minority of parental cells are expanded and become detectable as a consequence of iPSC generation (orange square). These variations are present in one allele of all the resulting iPSCs. Thus, their allele frequencies are expected to be ~50 % in iPSCs. (Middle) iPSC reprogramming per se introduces point mutations (star). These reprogramming-induced mutations can occur immediately after the onset of iPSC reprogramming (yellow star), which exhibit ~50 % allele frequencies. Furthermore, these mutations can occur after first- (red star) or second-cell division (green star) during iPSC reprogramming, which are expected to be observed at ~25 % or ~12.5 % allele frequencies, respectively. (Right) Mutations can arise during the prolonged culture (magenta circle), which can be observed at low allele frequencies

Because NGS can call genetic variations with their allele frequencies, several studies have attempted to elucidate the origin of these variations in iPSCs (Fig. 1). In the following section, we describe the origin of genetic variations in iPSCs.

## Origin of Genomic Instability in iPSCs

As mentioned earlier, genetic variations of iPSCs have at least three origins: (i) pre-existing variations in parental somatic cells, which can be manifested by cloning procedure during iPSC generation, (ii) reprogramming-induced mutations which occur during the reprogramming process, and (iii) passage-induced mutations which arise during the prolonged culture (Fig. 1).

### Pre-existing Variations in Parental Somatic Cells

Several studies employing NGS showed that a fraction of genetic variations found in iPSCs are present as pre-existing variations in parental somatic cells [31, 33, 34, 38, 39], and are fixed as a consequence of cloning process during iPSC generation. These studies performed sequencing analysis of iPSCs and their matched parental somatic cells to determine whether genetic variations originated from somatic cells. Furthermore, in another WGS study on mouse iPSCs, 157 shared SNVs were identified in four iPSC clones established from the same mouse embryonic fibroblasts (MEFs), which strongly suggests that these SNVs are most likely derived from their parental cells [40]. However, identification of pre-existing mutations is accompanied by technical difficulties as follows. Ultra-deep sequencing can be applied to search for pre-existing variations which exist at low frequencies in parental somatic cells [31, 34]. However, even when they are undetectable, it does not exclude the possibility that pre-existing variations might still exist at undetectably low frequencies. In addition, although NGS has the ability to detect low frequency variants, it is sometimes difficult to distinguish low frequency genuine biological variations from sequencing errors.

Two possible scenarios can be assumed with respect to pre-existing variations [32]. First, pre-existing variations are just randomly captured and expanded during the iPSC generation. Second, certain pre-existing variations can facilitate the reprogramming or proliferation of iPSCs, which could be preferentially propagated by selective advantage.

### Reprogramming-Induced Mutations

Ji et al. argued that 74 % of the point mutations were acquired during human iPSC reprogramming [35]. More recently, Sugiura et al. generated iPSC clones from MEFs prepared from embryo to minimize pre-existing mutations, and performed WGS analysis to reveal that hundreds of point mutations occur immediately after the onset of iPSC reprogramming. They also established subclones from an iPSC clone and confirmed the heterogeneity of point mutations within a single iPS clone, which indicated that these mutations were not derived from a parental cell. Furthermore, they established ESCs and iPSCs under nearly identical conditions and compared the point mutations profiles to demonstrate that the rate of point mutations in iPSCs were much higher than that in ESCs. This implicates that point mutations were introduced during reprogramming [41].

It is important to note the technical difficulties of distinguishing pre-existing variations and reprogramming-induced mutations. As shown in Fig. 1, pre-existing variations exist at ~50 % allele frequencies because they are present in one allele of all the iPSCs originated from a single parental cell. Meanwhile, as shown by Sugiura et al., reprogramming-induced mutations occur immediately after the onset of iPSC reprogramming (i.e., even before the first cell division or after the first-/second-cell division during iPSC reprogramming). Accordingly, these mutations can be observed at ~50 %, ~25 %, and ~12.5 % allele frequencies (Fig. 1) [41]. This indicates that SNVs with allele frequencies of ~50 % cannot be distinguished whether they are pre-existing variants or reprogramming-induced mutations solely based on allele frequencies. Interestingly, Sugiura et al. discovered that reprogramming-induced point mutations exhibit a transversion-dominant pattern, whereas pre-existing variations and passage-induced mutations exhibit a transition-dominant pattern [41]. The molecular mechanism by which reprogramming-induced mutations are introduced remains to be elucidated.

### Passage-Induced Mutations

Gore et al. applied WES for one human iPSC line at early and late passages, and demonstrated that four additional point mutations arose during the prolonged culture [34]. These mutations happen stochastically among cell population and are expected to exhibit lower allele frequencies (Fig. 1).

## Effects of Mutations on the Phenotype of iPSCs

Towards clinical applications, it is crucial to assess whether genetic variations in iPSCs can lead to unfavorable outcomes such as malignant outgrowth.

NGS technologies have enabled us to yield an unprecedented amount of information regarding cancer mutations [42, 43]. Exploration of cancer genomic data might provide an insight into the effect of genetic variations observed in iPSCs. Gore et al. pointed out that a majority of protein-coding mutations in iPSCs are nonsynonymous, nonsense, or splice variants, and are enriched in cancer-associated genes listed in the Catalogue of Somatic Mutations in Cancer (COSMIC) database [34, 44]. In contrast, a more recent study demonstrated that SNVs were not enriched in cancer-associated genes [33]. Importantly, Ruiz et al. assessed the functional effect of several protein-coding mutations identified in iPSCs on reprogramming efficiency by generating iPSCs that carry these mutations, and found that these mutations do not provide a selective advantage for reprogramming [36]. These two studies indicate that SNVs in iPSCs do not confer functional advantage by themselves.

The functional consequences of genetic variations need to be carefully interpreted. It is still difficult to distinguish “driver” mutations which confer a proliferative advantage contributing to cancer development from “passenger” mutations which have virtually no effect on the fitness of a cancer clone [45]. Hence, even when cancer mutations are found in iPSCs, it does not directly mean that these mutations lead to tumorigenesis. Validation experiments are a powerful method to confirm the functional effects of these mutations, but we have to bear in mind that a fraction of them are cell-type dependent (see Meyerson et al. [46] and Watson et al. [47] for review) Tumorigenic potential might also differ depending on the environment [48] surrounding the transplanted cells. Moreover, combination of mutation might lead to tumorigenic potential, as illustrated by multiple-hit hypothesis [49, 50]. Therefore, the phenotypic impact of genetic variations is sometimes hard to assess. However, it is noteworthy that donor-derived hematopoietic stem cells (HSCs) that harbored mutations in cancer-related *IDH2* and *DNMT3A* led to leukemia about two years after the transplantation [51]. Further studies are needed to investigate which genetic variations could confer harmful effects.

WGS analyses allow us to investigate mutations identified in non-coding regions in addition to coding mutations. Importantly, non-coding regions constitute around 98 % of the genome and contain a large number of *cis*-regulatory elements critical for regulation of gene expression [52]. Recently, cancer mutations have been identified in non-coding regulatory regions such as promoters and enhancers by WGS analyses [53–55]. Furthermore, disease-causative SNPs have been found to be overrepresented in non-coding enhancer regions [56] (for review see Murakawa et al. [57]), which highlights the importance of exploring mutations in non-coding regions. A recent WGS study of human iPSCs identified hundreds of mutations distributed throughout the genome [58]. These mutations were considered to be generally benign [58]. Further studies are need to better characterize non-coding mutations.

## Improvement of iPSC Generation Methods to Reduce Genomic Instability

Since the establishment of human iPSCs in 2007 [2, 3], many attempts have been made to produce iPSCs more efficiently and safely. Here we review recent papers with a particular emphasis on genomic instability.

### Starting Cell Source

It is important to consider the original source of somatic cells for iPSC generation. The first human iPSCs were generated from skin fibroblasts [2, 3], and since, skin-derived fibroblasts have been commonly used as a starting cell source. Although skin cells can be obtained more easily compared to other organ tissues, skin biopsies are still invasive. Meanwhile, a larger quantity of peripheral blood cells can be readily harvested. Peripheral blood mononuclear cells (PBMCs), as well as HSCs [59, 60], can be reprogrammed to iPSCs with high efficiency [61–63]. PBMC-derived iPSCs can be differentiated into mesenchymal stem cells, hepatocytes, and cardiomyocytes [64]. In addition, iPSCs can also be generated from cells isolated from urine [65], hair keratinocyte [66], mesenchymal stromal cells derived from wisdom teeth [67]. In the context of genomic instability, it was shown that protein-coding mutations were identified to a similar extent in human iPSCs derived from BJ fibroblasts, keratinocytes, mesenchymal stem cells, neural stem cells, and human umbilical vein endothelial cells [36].

Given that aging is associated with increased DNA damage [68], iPSCs derived from elderly patients might possess larger number of mutations. In fact, noncancerous skin cells from elderly subjects harbored a comparable number of somatic mutations to that in skin cancer cells and a fraction of these mutations were identified in cancer-associated genes [69]. Somatic mutations, including cancer driver mutations, have been shown to accumulate in blood cells with increasing age [70, 71]. More recently, it was demonstrated that mitochondrial DNA (mtDNA) mutations in human iPSCs increased with age, which compromised the metabolic function in iPSCs [72]. It was also reported that iPSCs derived from older mice exhibited lower proliferative activity and reprogramming efficiency [73]. These findings suggest that cells from younger donors may be advantageous.

Umbilical cord blood cells can be collected non-invasively from the umbilical cord at the time of birth. Umbilical cord blood cells contain hematopoietic stem and progenitor cells, and are banked together with immunological information for the treatment of hematological malignancies [74]. In addition, iPSCs have been successfully generated from umbilical cord blood cells [63, 75, 76]. Notably, a WES study revealed that umbilical cord blood-derived iPSCs harbored remarkably lower point mutations than fibroblast-derived iPSCs [77].

These days iPSC-based cell therapies are switching from autologous transplantation to allogeneic transplantation [78]. Although immune rejection can be avoided in autologous transplantation of patient-matched iPSCs [6, 7], generation of patient-derived iPSCs is a time-consuming and expensive processes [79]. Thus, autologous transplantation cannot be readily applicable for acute progressive disorders. Importantly, a small number of homozygous human leukocyte antigen (HLA) types could cover a large portion of populations [80, 81], making them ideal biological resources for allogeneic transplantation [79]. Considering the lower mutational load in umbilical cord blood cells and the availability of immunological information, HLA-matched umbilical cord blood-derived iPSCs are potentially ideal cell sources for allogenic iPSC-based cell therapies [82]. It would be important to bank iPSCs together with their genomic data [79, 83] because the data can be used to study the effect of genetic variations on clinical outcome of iPSC-based transplantation and help formulate evidence-based criteria for clinical applications.

### Delivery Method

A number of studies have aimed to improve the efficiency and safety of iPSC reprogramming. Originally, iPSCs were generated using retroviruses [1–3]. However, integrated viral genome could produce insertional mutations and reactivate transgenes after reprogramming [84], which might play a role in tumorigenesis. Indeed, reactivation of c-Myc transgene caused tumors in mouse iPSCs [85]. To circumvent this problem, integration-free vectors such as expression plasmids [86], Sendai virus vectors [87], and episomal plasmid vectors [88, 89] have been developed. In addition, several DNA-free reprogramming methods such as protein-based methods [90, 91] or mRNA-based methods [92] have also been developed.

Several studies have compared genomic instabilities in iPSCs generated via different methods. Gore et al. [34] and Bhutani et al. [58] have demonstrated that the numbers of SNVs were comparable between different reprogramming methods. However, Sugiura et al. showed that retrovirally transduced iPSCs harbored about twice as many mutations as integration-free iPSCs [41]. Cheng et al. reported that incidence of genetic variations were low in human iPSCs generated by nonintegrating plasmid expression method [33]. These two studies conclude that integration-free delivery methods are currently most effective and might be ideal for clinical applications.

### Reprogramming Factor

In addition to delivery methods, reprogramming factors have been explored to generate safer iPSCs more effectively. Recently, NuRD (nucleosome remodeling and deacetylation) component Mbd3 has been identified as a major reprogramming barrier during iPSC induction [93]. In fact, depletion of Mbd3 significantly increased the efficiency of iPSC reprogramming [93]. Moreover, oocyte factor Zspan4 improves not only reprogramming efficiency but also genomic stability during mouse iPSC reprogramming [94]. A more recent study revealed that reduction of replication stress during reprogramming by overexpressing checkpoint kinase 1 (CHK1) increases the iPSC reprogramming efficiency and genomic stability in both mouse and human [95].

Reprogramming factors which are currently used, such as OCT4, SOX2, KLF4, c-MYC, NANOG and LIN28, are reported to have oncogenic potential [96–102]. Given that such pluripotency-associated genes can lead to tumorigenesis, chemical induction might help reduce the risk of tumorigenesis. Notably, Hou et al. succeeded in generating iPSCs with a combination of seven small-molecule compounds [103].

### Alternative Reprogramming Method

Back in 1962, Gurdon succeeded in generating cloned frogs by transferring the nucleus of a differentiated tadpole's somatic cell into an oocyte [104]. This method is referred to as somatic cell nuclear transfer (SCNT). Recently, human SCNT-ESCs were successfully generated from adult somatic cells [105], and several genome-wide analyses have been performed for iPSCs and SCNT-ESCs derived from the genetically matched somatic cells [37, 106]. Reprogramming process has been reported to be immediate in human SCNT-ESCs but gradual in human iPSCs [107], suggesting that mutational processes might be distinct. However, human SCNT-ESCs and iPSCs contained similar levels of CNVs [106] and protein-coding mutations [37]. Meanwhile, a potential advantage of SCNT-ESCs is that SCNT technology can rescue the mtDNA mutations by replacing old somatic mitochondria with oocyte mitochondria [72]. However, SCNT-ESCs are technically challenging and pose several ethical issues.

### Cell Passage

As we described earlier, deleterious CNVs which occurr at earlier passages could be negatively selected and lost during subsequent passages [30]. However, several studies reported that aneuploidies [19, 108], CNVs [22, 108], and point mutations [34] accumulate at later passages. Further studies are required to determine the optimal passage number for clinical use.

## Concluding Remarks and Future Directions

Genomic instability can occur at any stage of iPSC generation. Mutations could also arise during differentiation of iPSCs to final cell products to be used for transplantation. Taking into account genomic instability, malignant outgrowth can be of serious concern. Therefore, careful monitoring is crucial to ensure iPSC safety prior to clinical applications [34]. However, even though NGS technologies have significantly reduced in cost [109], extensive genome-wide analysis on a routine basis is still financially inefficient. Large-scale WGS studies of iPSCs might lead to the identification of genetic variations which are relevant to clinical outcome, resulting in cost-effective and target-specific analysis. Considering the current limitations of comprehensive genetic testing, tumor formation assay might be one way of assessing the tumorigenic potential of iPSC-derived products [110]. However, there are currently no evidence-based guidelines for tumorigenicity testing of iPSC-derived cell products. Recently, it has been reported that human iPSC-derived neurospheres formed tumors in a mouse model after long-term observation [111], indicating the importance of long-term follow up. In the case of iPSC-derived RPE cell transplantation, ocular fundus can be observed noninvasively [10], and morphological changes of transplanted RPE can be monitored at cellular levels using optical coherence tomography [112, 113]. In addition to genomic instability, contamination of residual undifferentiated iPSCs or residual exogenous genes could play a role in tumorigenesis after transplantation [114]. Several strategies have been developed to prevent teratoma formation. Residual cells can be eliminated by immunodepletion using antibodies against stage-specific embryonic antigen-5 (SSEA-5) and two additional surface proteins related to pluripotency [115], or through small chemical molecules [114].

Here we reviewed recent works describing genomic instability in iPSCs in the context of clinical applications. Currently only a limited number of genome-wide studies of iPSCs have been conducted as described here. In the near future, iPSC-based cell therapies can be expected to be applied to many diseases involving other organs such as liver [116], kidney [117], and cornea [118] (for review see Okano & Yamanaka [119]). To ensure the safety of forthcoming iPSC-derived novel therapies, a more comprehensive understanding of genetic variations in the genome of iPSCs is important, and validation experiments are necessary to identify functional consequence of genetic variations. Moreover, so far there has been only one clinical trial of iPSC-based therapy [8], which limits the assessment of safety issues.

Since the first establishment of human iPSCs in 2007, many improvements have been made to increase the safety and efficiency of iPSCs. Genomic and functional evaluation of iPSCs would be important with the advent of newer iPSC generation protocols. Furthermore, better understanding of the mechanism underlying genetic variations in iPSCs will help to reduce genetic variations in iPSCs. Epigenomic instability could also be considered (see Lund et al. [25] and Liang & Zhang [32] for review). In summary, better characterization of iPSCs will pave the way for clinical applications of iPSC-based cell therapies.

## References

[1] Takahashi K, Yamanaka S (2006). Induction of pluripotent stem cells from mouse embryonic and adult fibroblast cultures by defined factors. Cell.

[2] Yu J, Vodyanik MA, Smuga-Otto K (2007). Induced pluripotent stem cell lines derived from human somatic cells. Science.

[3] Takahashi K, Tanabe K, Ohnuki M (2007). Induction of pluripotent stem cells from adult human fibroblasts by defined factors. Cell.

[4] Avior Y, Sagi I, Benvenisty N (2016). Pluripotent stem cells in disease modelling and drug discovery. Nature Reviews. Molecular Cell Biology.

[5] Robinton DA, Daley GQ (2012). The promise of induced pluripotent stem cells in research and therapy. Nature.

[6] Araki R, Uda M, Hoki Y (2013). Negligible immunogenicity of terminally differentiated cells derived from induced pluripotent or embryonic stem cells. Nature.

[7] Guha P, Morgan JW, Mostoslavsky G, Rodrigues NP, Boyd AS (2013). Lack of immune response to differentiated cells derived from syngeneic induced pluripotent stem cells. Cell Stem Cell.

[8] Cyranoski D (2014). Japanese woman is first recipient of next-generation stem cells. Nature.

[9] Chakradhar S (2016). An eye to the future: Researchers debate best path for stem cell-derived therapies. Nature Medicine.

[10] Garber K (2015). RIKEN suspends first clinical trial involving induced pluripotent stem cells. Nature Biotechnology.

[11] Yunis JJ (1976). High resolution of human chromosomes. Science.

[12] Meisner LF, Johnson JA (2008). Protocols for cytogenetic studies of human embryonic stem cells. Methods.

[13] Kallioniemi A, Kallioniemi OP, Sudar D (1992). Comparative genomic hybridization for molecular cytogenetic analysis of solid tumors. Science.

[14] Wang DG, Fan JB, Siao CJ (1998). Large-scale identification, mapping, and genotyping of single-nucleotide polymorphisms in the human genome. Science.

[15] Le Scouarnec S, Gribble SM (2012). Characterising chromosome rearrangements: recent technical advances in molecular cytogenetics. Heredity.

[16] Riegel M (2014). Human molecular cytogenetics: from cells to nucleotides. Genetics and Molecular Biology.

[17] Metzker ML (2010). Sequencing technologies - the next generation. Nature Reviews. Genetics.

[18] Pagnamenta AT, Lise S, Harrison V (2012). Exome sequencing can detect pathogenic mosaic mutations present at low allele frequencies. Journal of Human Genetics.

[19] Mayshar Y, Ben-David U, Lavon N (2010). Identification and classification of chromosomal aberrations in human induced pluripotent stem cells. Cell Stem Cell.

[20] Taapken SM, Nisler BS, Newton MA (2011). Karotypic abnormalities in human induced pluripotent stem cells and embryonic stem cells. Nature Biotechnology.

[21] Martins-Taylor K, Nisler BS, Taapken SM (2011). Recurrent copy number variations in human induced pluripotent stem cells. Nature Biotechnology.

[22] Laurent LC, Ulitsky I, Slavin I (2011). Dynamic changes in the copy number of pluripotency and cell proliferation genes in human ESCs and iPSCs during reprogramming and time in culture. Cell Stem Cell.

[23] McIntyre A, Summersgill B, Lu YJ (2007). Genomic copy number and expression patterns in testicular germ cell tumours. British Journal of Cancer.

[24] Reuter VE (2005). Origins and molecular biology of testicular germ cell tumors. Modern Pathology.

[25] Lund RJ, Narva E, Lahesmaa R (2012). Genetic and epigenetic stability of human pluripotent stem cells. Nature Reviews Genetics.

[26] Chin MH, Mason MJ, Xie W (2009). Induced pluripotent stem cells and embryonic stem cells are distinguished by gene expression signatures. Cell Stem Cell.

[27] Elliott AM, Elliott KA, Kammesheidt A (2010). High resolution array-CGH characterization of human stem cells using a stem cell focused microarray. Molecular Biotechnology.

[28] Guled M, Myllykangas S, Frierson HF, Mills SE, Knuutila S, Stelow EB (2008). Array comparative genomic hybridization analysis of olfactory neuroblastoma. Modern Pathology.

[29] Scotto L, Narayan G, Nandula SV (2008). Identification of copy number gain and overexpressed genes on chromosome arm 20q by an integrative genomic approach in cervical cancer: potential role in progression. Genes, Chromosomes and Cancer.

[30] Hussein SM, Batada NN, Vuoristo S (2011). Copy number variation and selection during reprogramming to pluripotency. Nature.

[31] Abyzov A, Mariani J, Palejev D (2012). Somatic copy number mosaicism in human skin revealed by induced pluripotent stem cells. Nature.

[32] Liang G, Zhang Y (2013). Genetic and epigenetic variations in iPSCs: potential causes and implications for application. Cell Stem Cell.

[33] Cheng L, Hansen NF, Zhao L (2012). Low incidence of DNA sequence variation in human induced pluripotent stem cells generated by nonintegrating plasmid expression. Cell Stem Cell.

[34] Gore A, Li Z, Fung HL (2011). Somatic coding mutations in human induced pluripotent stem cells. Nature.

[35] Ji J, Ng SH, Sharma V (2012). Elevated coding mutation rate during the reprogramming of human somatic cells into induced pluripotent stem cells. Stem Cells.

[36] Ruiz S, Gore A, Li Z (2013). Analysis of protein-coding mutations in hiPSCs and their possible role during somatic cell reprogramming. Nature Communications.

[37] Johannesson B, Sagi I, Gore A (2014). Comparable frequencies of coding mutations and loss of imprinting in human pluripotent cells derived by nuclear transfer and defined factors. Cell Stem Cell.

[38] Quinlan AR, Boland MJ, Leibowitz ML (2011). Genome sequencing of mouse induced pluripotent stem cells reveals retroelement stability and infrequent DNA rearrangement during reprogramming. Cell Stem Cell.

[39] Howden SE, Gore A, Li Z (2011). Genetic correction and analysis of induced pluripotent stem cells from a patient with gyrate atrophy. Proceedings of the National Academy of Sciences of the United States of America.

[40] Young MA, Larson DE, Sun CW (2012). Background mutations in parental cells account for most of the genetic heterogeneity of induced pluripotent stem cells. Cell Stem Cell.

[41] Sugiura M, Kasama Y, Araki R (2014). Induced pluripotent stem cell generation-associated point mutations arise during the initial stages of the conversion of these cells. Stem Cell Reports.

[42] Hudson TJ, Anderson W, Artez A (2010). International network of cancer genome projects. Nature.

[43] Weinstein JN, Collisson EA, Mills GB (2013). The cancer genome atlas Pan-cancer analysis project. Nature Genetics.

[44] Forbes SA, Beare D, Gunasekaran P (2015). COSMIC: exploring the world's knowledge of somatic mutations in human cancer. Nucleic Acids Research.

[45] Gonzalez-Perez A, Mustonen V, Reva B (2013). Computational approaches to identify functional genetic variants in cancer genomes. Nature Methods.

[46] Meyerson M, Gabriel S, Getz G (2010). Advances in understanding cancer genomes through second-generation sequencing. Nature Reviews Genetics.

[47] Watson IR, Takahashi K, Futreal PA, Chin L (2013). Emerging patterns of somatic mutations in cancer. Nature Reviews Genetics.

[48] Fidler IJ (2003). The pathogenesis of cancer metastasis: the 'seed and soil' hypothesis revisited. Nature Reviews. Cancer.

[49] Knudson AG (1971). Mutation and cancer: statistical study of retinoblastoma. Proceedings of the National Academy of Sciences of the United States of America.

[50] Berger AH, Knudson AG, Pandolfi PP (2011). A continuum model for tumour suppression. Nature.

[51] Yasuda T, Ueno T, Fukumura K (2014). Leukemic evolution of donor-derived cells harboring IDH2 and DNMT3A mutations after allogeneic stem cell transplantation. Leukemia.

[52] Consortium E.P (2012). An integrated encyclopedia of DNA elements in the human genome. Nature.

[53] Fredriksson NJ, Ny L, Nilsson JA, Larsson E (2014). Systematic analysis of noncoding somatic mutations and gene expression alterations across 14 tumor types. Nature Genetics.

[54] Weinhold N, Jacobsen A, Schultz N, Sander C, Lee W (2014). Genome-wide analysis of noncoding regulatory mutations in cancer. Nature Genetics.

[55] Melton C, Reuter JA, Spacek DV, Snyder M (2015). Recurrent somatic mutations in regulatory regions of human cancer genomes. Nature Genetics.

[56] Farh KK, Marson A, Zhu J (2015). Genetic and epigenetic fine mapping of causal autoimmune disease variants. Nature.

[57] Murakawa Y, Yoshihara M, Kawaji H (2016). Enhanced identification of transcriptional enhancers provides mechanistic insights into diseases. Trends in Genetics.

[58] Bhutani K, Nazor KL, Williams R (2016). Whole-genome mutational burden analysis of three pluripotency induction methods. Nature Communications.

[59] Loh YH, Agarwal S, Park IH (2009). Generation of induced pluripotent stem cells from human blood. Blood.

[60] Merling RK, Sweeney CL, Choi U (2013). Transgene-free iPSCs generated from small volume peripheral blood nonmobilized CD34+ cells. Blood.

[61] Ye Z, Zhan H, Mali P (2009). Human-induced pluripotent stem cells from blood cells of healthy donors and patients with acquired blood disorders. Blood.

[62] Staerk J, Dawlaty MM, Gao Q (2010). Reprogramming of human peripheral blood cells to induced pluripotent stem cells. Cell Stem Cell.

[63] Okita K, Yamakawa T, Matsumura Y (2013). An efficient nonviral method to generate integration-free human-induced pluripotent stem cells from cord blood and peripheral blood cells. Stem Cells.

[64] Su RJ, Baylink DJ, Neises A (2013). Efficient generation of integration-free ips cells from human adult peripheral blood using BCL-XL together with Yamanaka factors. PLoS One.

[65] Xue Y, Cai X, Wang L (2013). Generating a non-integrating human induced pluripotent stem cell bank from urine-derived cells. PLoS One.

[66] Aasen T, Raya A, Barrero MJ (2008). Efficient and rapid generation of induced pluripotent stem cells from human keratinocytes. Nature Biotechnology.

[67] Oda Y, Yoshimura Y, Ohnishi H (2010). Induction of pluripotent stem cells from human third molar mesenchymal stromal cells. Journal of Biological Chemistry.

[68] Garinis GA, van der Horst GT, Vijg J, Hoeijmakers JH (2008). DNA damage and ageing: new-age ideas for an age-old problem. Nature Cell Biology.

[69] Martincorena I, Roshan A, Gerstung M (2015). Tumor evolution. High burden and pervasive positive selection of somatic mutations in normal human skin. Science.

[70] Jaiswal S, Fontanillas P, Flannick J (2014). Age-related clonal hematopoiesis associated with adverse outcomes. New England Journal of Medicine.

[71] Genovese G, Kahler AK, Handsaker RE (2014). Clonal hematopoiesis and blood-cancer risk inferred from blood DNA sequence. New England Journal of Medicine.

[72] Kang E, Wang X, Tippner-Hedges R (2016). Age-related accumulation of somatic mitochondrial DNA mutations in adult-derived human iPSCs. Cell Stem Cell.

[73] Wang B, Miyagoe-Suzuki Y, Yada E (2011). Reprogramming efficiency and quality of induced Pluripotent Stem Cells (iPSCs) generated from muscle-derived fibroblasts of mdx mice at different ages. PLoS Currents.

[74] Rocha V, Labopin M, Sanz G (2004). Transplants of umbilical-cord blood or bone marrow from unrelated donors in adults with acute leukemia. New England Journal of Medicine.

[75] Haase A, Olmer R, Schwanke K (2009). Generation of induced pluripotent stem cells from human cord blood. Cell Stem Cell.

[76] Giorgetti A, Montserrat N, Aasen T (2009). Generation of induced pluripotent stem cells from human cord blood using OCT4 and SOX2. Cell Stem Cell.

[77] Su RJ, Yang Y, Neises A (2013). Few single nucleotide variations in exomes of human cord blood induced pluripotent stem cells. PLoS One.

[78] Blair NF, Barker RA (2016). Making it personal: the prospects for autologous pluripotent stem cell-derived therapies. Regenerative Medicine.

[79] de Rham C, Villard J (2014). Potential and limitation of HLA-based banking of human pluripotent stem cells for cell therapy. Journal of Immunology Research.

[80] Taylor CJ, Peacock S, Chaudhry AN, Bradley JA, Bolton EM (2012). Generating an iPSC bank for HLA-matched tissue transplantation based on known donor and recipient HLA types. Cell Stem Cell.

[81] Nakajima F, Tokunaga K, Nakatsuji N (2007). Human leukocyte antigen matching estimations in a hypothetical bank of human embryonic stem cell lines in the Japanese population for use in cell transplantation therapy. Stem Cells.

[82] Broxmeyer HE (2010). Will iPS cells enhance therapeutic applicability of cord blood cells and banking?. Cell Stem Cell.

[83] Solomon S, Pitossi F, Rao MS (2015). Banking on iPSC--is it doable and is it worthwhile. Stem Cell Reviews.

[84] Baum C, von Kalle C, Staal FJ (2004). Chance or necessity? Insertional mutagenesis in gene therapy and its consequences. Molecular Therapy.

[85] Okita K, Ichisaka T, Yamanaka S (2007). Generation of germline-competent induced pluripotent stem cells. Nature.

[86] Okita K, Nakagawa M, Hyenjong H, Ichisaka T, Yamanaka S (2008). Generation of mouse induced pluripotent stem cells without viral vectors. Science.

[87] Fusaki N, Ban H, Nishiyama A, Saeki K, Hasegawa M (2009). Efficient induction of transgene-free human pluripotent stem cells using a vector based on Sendai virus, an RNA virus that does not integrate into the host genome. Proceedings of the Japan Academy. Series B, Physical and Biological Sciences.

[88] Yu J, Hu K, Smuga-Otto K (2009). Human induced pluripotent stem cells free of vector and transgene sequences. Science.

[89] Okita K, Matsumura Y, Sato Y (2011). A more efficient method to generate integration-free human iPS cells. Nature Methods.

[90] Zhou H, Wu S, Joo JY (2009). Generation of induced pluripotent stem cells using recombinant proteins. Cell Stem Cell.

[91] Kim D, Kim CH, Moon JI (2009). Generation of human induced pluripotent stem cells by direct delivery of reprogramming proteins. Cell Stem Cell.

[92] Warren L, Manos PD, Ahfeldt T (2010). Highly efficient reprogramming to pluripotency and directed differentiation of human cells with synthetic modified mRNA. Cell Stem Cell.

[93] Rais Y, Zviran A, Geula S (2013). Deterministic direct reprogramming of somatic cells to pluripotency. Nature.

[94] Jiang J, Lv W, Ye X (2013). Zscan4 promotes genomic stability during reprogramming and dramatically improves the quality of iPS cells as demonstrated by tetraploid complementation. Cell Research.

[95] Ruiz S, Lopez-Contreras AJ, Gabut M (2015). Limiting replication stress during somatic cell reprogramming reduces genomic instability in induced pluripotent stem cells. Nature Communications.

[96] Gidekel S, Pizov G, Bergman Y, Pikarsky E (2003). Oct-3/4 is a dose-dependent oncogenic fate determinant. Cancer Cell.

[97] Almstrup K, Hoei-Hansen CE, Wirkner U (2004). Embryonic stem cell-like features of testicular carcinoma in situ revealed by genome-wide gene expression profiling. Cancer Research.

[98] Boumahdi S, Driessens G, Lapouge G (2014). SOX2 controls tumour initiation and cancer stem-cell functions in squamous-cell carcinoma. Nature.

[99] Rowland BD, Peeper DS (2006). KLF4, p21 and context-dependent opposing forces in cancer. Nature Reviews. Cancer.

[100] Pelengaris S, Khan M, Evan G (2002). c-MYC: more than just a matter of life and death. Nature Reviews. Cancer.

[101] Hart AH, Hartley L, Parker K (2005). The pluripotency homeobox gene NANOG is expressed in human germ cell tumors. Cancer.

[102] West JA, Viswanathan SR, Yabuuchi A (2009). A role for Lin28 in primordial germ-cell development and germ-cell malignancy. Nature.

[103] Hou P, Li Y, Zhang X (2013). Pluripotent stem cells induced from mouse somatic cells by small-molecule compounds. Science.

[104] Gurdon JB (1962). Adult frogs derived from the nuclei of single somatic cells. Developmental Biology.

[105] Chung YG, Eum JH, Lee JE (2014). Human somatic cell nuclear transfer using adult cells. Cell Stem Cell.

[106] Ma H, Morey R, O'Neil RC (2014). Abnormalities in human pluripotent cells due to reprogramming mechanisms. Nature.

[107] Egli D, Chen AE, Saphier G (2011). Reprogramming within hours following nuclear transfer into mouse but not human zygotes. Nature Communications.

[108] Liu P, Kaplan A, Yuan B, Hanna JH, Lupski JR, Reiner O (2014). Passage number is a major contributor to genomic structural variations in mouse iPSCs. Stem Cells.

[109] Goodwin S, McPherson JD, McCombie WR (2016). Coming of age: ten years of next-generation sequencing technologies. Nature Reviews Genetics.

[110] Kawamata S, Kanemura H, Sakai N, Takahashi M, Go MJ (2015). Design of a tumorigenicity test for induced pluripotent stem cell (iPSC)-derived cell products. Journal of Clinical Medicine.

[111] Nori S, Okada Y, Nishimura S (2015). Long-term safety issues of iPSC-based cell therapy in a spinal cord injury model: oncogenic transformation with epithelial-mesenchymal transition. Stem Cell Reports.

[112] Jaffe GJ, Caprioli J (2004). Optical coherence tomography to detect and manage retinal disease and glaucoma. American Journal of Ophthalmology.

[113] Fujimoto JG, Pitris C, Boppart SA, Brezinski ME (2000). Optical coherence tomography: an emerging technology for biomedical imaging and optical biopsy. Neoplasia (New York, NY).

[114] Lee MO, Moon SH, Jeong HC (2013). Inhibition of pluripotent stem cell-derived teratoma formation by small molecules. Proceedings of the National Academy of Sciences of the United States of America.

[115] Tang C, Lee AS, Volkmer JP (2011). An antibody against SSEA-5 glycan on human pluripotent stem cells enables removal of teratoma-forming cells. Nature Biotechnology.

[116] Takebe T, Sekine K, Enomura M (2013). Vascularized and functional human liver from an iPSC-derived organ bud transplant. Nature.

[117] Taguchi A, Kaku Y, Ohmori T (2014). Redefining the in vivo origin of metanephric nephron progenitors enables generation of complex kidney structures from pluripotent stem cells. Cell Stem Cell.

[118] Hayashi R, Ishikawa Y, Sasamoto Y (2016). Co-ordinated ocular development from human iPS cells and recovery of corneal function. Nature.

[119] Okano H, Yamanaka S (2014). iPS cell technologies: significance and applications to CNS regeneration and disease. Molecular Brain.

